# Real world evidence of calcifediol or vitamin D prescription and mortality rate of COVID-19 in a retrospective cohort of hospitalized Andalusian patients

**DOI:** 10.1038/s41598-021-02701-5

**Published:** 2021-12-03

**Authors:** Carlos Loucera, María Peña-Chilet, Marina Esteban-Medina, Dolores Muñoyerro-Muñiz, Román Villegas, Jose Lopez-Miranda, Jesus Rodriguez-Baño, Isaac Túnez, Roger Bouillon, Joaquin Dopazo, Jose Manuel Quesada Gomez

**Affiliations:** 1 Clinical Bioinformatics Area, Fundación Progreso y Salud (FPS), CDCA, Hospital Virgen del Rocio, 41013 Seville, Spain; 2 Institute of Biomedicine of Seville (IBIS), Hospital Virgen del Rocio, 41013 Seville, Spain; 3 Bioinformatics in Rare Diseases (BiER), Centro de Investigación Biomédica en Red de Enfermedades Raras (CIBERER), FPS, Hospital Virgen del Rocio, 41013 Seville, Spain; 4 Subdirección Técnica Asesora de Gestión de la Información, Servicio Andaluz de Salud, Seville, Spain; 5 Internal Medicine Department, IMIBIC/Reina Sofia University Hospital/University of Cordoba, 14004 Córdoba, Spain; 6 CIBER Fisiopatologia Obesidad y Nutricion (CIBEROBN), Instituto de Salud Carlos III, 28029 Madrid, Spain; 7 Unidad Clínica de Enfermedades Infecciosas, Microbiología y Medicina Preventiva, Hospital Universitario Virgen Macarena, Seville, Spain; 8 Departamento de Medicina, Universidad de Sevilla, Seville, Spain; 9 Departamento de Bioquimica y Biología Molecular, Facultad de Medicina y Enfermería, Universidad de Córdoba, Córdoba, Spain; 10 Instituto Maimónides de Investigacion Biomédica de Córdoba (IMIBIC), 14004 Córdoba, Spain; 11 G. Técnico de Expertos de Andalucía para Estudios de Suplementos e Intervención Nutricional Frente a Covid-19, SGIDIS, Consejería de Salud y Familias, Junta de Andalucia, Seville, Spain; 12 Secretaria General de Investigación, Desarrollo e Innovación en Salud, Consejería de Salud y Familias de la Junta de Andalucía, Seville, Spain; 13 Clinical and Experimental Endocrinology, Department of Chronic Diseases and Metabolism, KULeuven, Herestraat, 3000 Leuven, Belgium; 14 FPS/ELIXIR-ES, Fundación Progreso y Salud (FPS), CDCA, Hospital Virgen del Rocio, 41013 Seville, Spain; 15 CIBER de Fragilidad y Envejecimiento Saludable (CIBERFES), Hospital Universitario Reina Sofía, Universidad de Córdoba, Menéndez Pidal s/n, 14004 Córdoba, Spain

**Keywords:** Biochemistry, Computational biology and bioinformatics, Drug discovery, Endocrinology, Risk factors

## Abstract

COVID-19 is a major worldwide health problem because of acute respiratory distress syndrome, and mortality. Several lines of evidence have suggested a relationship between the vitamin D endocrine system and severity of COVID-19. We present a survival study on a retrospective cohort of 15,968 patients, comprising all COVID-19 patients hospitalized in Andalusia between January and November 2020. Based on a central registry of electronic health records (the Andalusian Population Health Database, BPS), prescription of vitamin D or its metabolites within 15–30 days before hospitalization were recorded. The effect of prescription of vitamin D (metabolites) for other indication previous to the hospitalization was studied with respect to patient survival. Kaplan–Meier survival curves and hazard ratios support an association between prescription of these metabolites and patient survival. Such association was stronger for calcifediol (Hazard Ratio, HR = 0.67, with 95% confidence interval, CI, of [0.50–0.91]) than for cholecalciferol (HR = 0.75, with 95% CI of [0.61–0.91]), when prescribed 15 days prior hospitalization. Although the relation is maintained, there is a general decrease of this effect when a longer period of 30 days prior hospitalization is considered (calcifediol HR = 0.73, with 95% CI [0.57–0.95] and cholecalciferol HR = 0.88, with 95% CI [0.75, 1.03]), suggesting that association was stronger when the prescription was closer to the hospitalization.

## Introduction

Vitamin D deficiency has been associated with a large number of diseases including immune disorders and infections. The causal role of vitamin D for rickets and osteomalacia is well demonstrated and its role in aggravating osteoporosis is well accepted^1^. Vitamin D3 (cholecalciferol), the threshold nutrient of the vitamin D endocrine system (VDES), is acquired by cutaneous synthesis under the influence of UV-B light and in minimal amounts from the diet. It is transported, like other VDES metabolites, by vitamin D-binding protein (*DBP*). Vitamin D is converted to 25-hydroxyvitamin D (25OHD) in the liver, through the action of 25 hydroxylase (mainly *CYP2R1* and some other P450 enzymes). 25OHD or calcifediol is the best biomarker of nutritional status. It is also is substrate for the synthesis of 1,25(OH)2D or calcitriol through the action of 1α hydroxylase (*CYP27B*) in the kidney for its endocrine actions, and in multiple cells of the body for its auto/paracrine action. The systemic hormone calcitriol binds with high affinity to its nuclear receptor, the vitamin D receptor (*VDR*), regulating transcription of a large number (~ 3%) of genes, with a broad spectrum of functional activities^1^. However, its extra-skeletal effects are more disputed. Several Mendelian Randomization studies demonstrated that genetically low serum 25OHD concentrations increase the risk of multiple sclerosis^2^ and a meta-analysis found a reduced incidence of upper respiratory infections when supplements of vitamin D are given to relatively vitamin D deficient subjects^3^. There are now multiple association studies^4–10^ and meta-analyses^11–13^ linking a poor vitamin D status with increased risk^4,8–10^ or severity of COVID-19 infections^4–7^.

A recent UK study by NICE, however, concluded that there is insufficient evidence to recommend vitamin D supplementation solely for the purpose of prevention of COVID-19 (complications) but recommends the UK guidelines to prevent vitamin D deficiency in general. The NICE experts agreed that a poor vitamin D status was associated with more severe outcomes from COVID-19 but without proof of causality, especially because the risk factors for severe COVID-19 outcomes are also risk factors for low vitamin D status^14^. This is also the conclusion from a recent Cochrane analysis^15^. Moreover, a recent study on systematic drug repurposing for COVID-19 based on machine learning has found that, among others, the *VDR* protein could have a protector effect over pathways affected by the SARS-CoV-2 infection^16^, suggesting a potential protecting role for VDES metabolites such as cholecalciferol, calcifediol or calcitriol. This drug repurposing study used mechanistic models^17^ of the COVID-19 disease map^18^ to find relevant interactions between proteins (already targets of drugs with other indications) and the pathways affected by COVID-19 disease infection either directly or downstream, collectively known as the COVID-19 disease map^18^, thus providing mechanistic evidences of the protective effect of VDES metabolites in COVID-19.

Although randomized clinical trials remain the gold standard to prove efficacy and safety of whatever interventions^19^, other types of studies may be faster and more efficient to provide clinical guidelines, especially when lifesaving procedures are needed in an emergency situation such as the present COVID-19 pandemic. Thus, the increasing availability of digital health data, together with the raising costs and known limitations of traditional trials, has fostered the interest in the use of real-world data (RWD)^20^, defined as patient’s data on their health status and on health care received, collected from their electronic health records (EHR)^20,21^. RWD can be analyzed to generate real word evidence (RWE)^22^. Actually, RWE provide a better image of the actual clinical environments in which medical interventions are carried out when compared to conventional randomized clinical trials, given that RWD includes detailed data on patient demographics, comorbidities, adherence, and simultaneous prescriptions^23,24^.

Since 2001, the Andalusian Public Health System has been thoroughly storing all the EHRs data of Andalusian patients in the Population Health Base (BPS)^25^. This makes of BPS one of the largest repositories of highly detailed clinical data in the world (with over 13 million of comprehensive registries)^25^. BPS constitutes a unique and privileged environment to carry out large-scale RWE studies.

Here we used RWD from BPS to obtain RWE of the effectiveness of the prior prescription of cholecalciferol, calcifediol or calcitriol VDES metabolites with nutrient, pre-hormone or hormone activity respectively, on mortality rate among patients hospitalized for COVID-19.

## Results

### Data processing

A retrospective cohort of 15,968 patients, which include all Andalusian patients with COVID-19 diagnosis that were hospitalized between January and November 2020, was found in BPS and collected. Figure S1 depicts the frequency of hospital admission of patients with COVID-19 diagnosis along this period. Patient data on medication and other relevant covariates (see Table 1) was downloaded from the BPS.

**Table 1:** Data imported from BPS for each patient: code and definition of the variable.

Code	Meaning
FECNAC	Birth date
FECDEF	Death date
SEXO	Gender
FEC_INGRESO	Hospital admission date
FEC_ALTA	Discharge date
MOTIVO_ALTA	Reason for the discharge: (recovery/death/admission in another hospital/voluntary discharge/retirement home/unspecified)
COD_PATOLOGIA_CRONICA	Hospital codes for chronic conditions
COD_FEC_INI_PATOLOGIA	Date of condition diagnosis
COD_CIE_NORMALIZADO	A mixture of ICD9 and ICD10 codes for diseases
DESC_CIE_NORMALIZADO	Description of the ICD
FECINI_DIAG	Diagnosis date
FECFIN_DIAG	End of the diagnosed condition
FUENTE_DIAG	Source of the diagnosis (hospital, emergency, etc.)
IND_CRONICO_HCUP	Is a chronic disease? (yes/no)
Test COVID: FECHA	Test COVID date
Test COVID: TYPE	PCR/antigens
Test COVID: RESULTADO_TEST	Result of the test (positive/negative)
Pharmacy (Hospital and external): DESCRIPCION	List of drugs used in hospital or purchased in the pharmacies
Pharmacy (Hospital and external): FECHA	Dispensing date
VACUNA	List of vaccines
VACUNAFECHA	Vaccination dates

### Vitamin D endocrine system metabolites and survival

**Figure 1:**
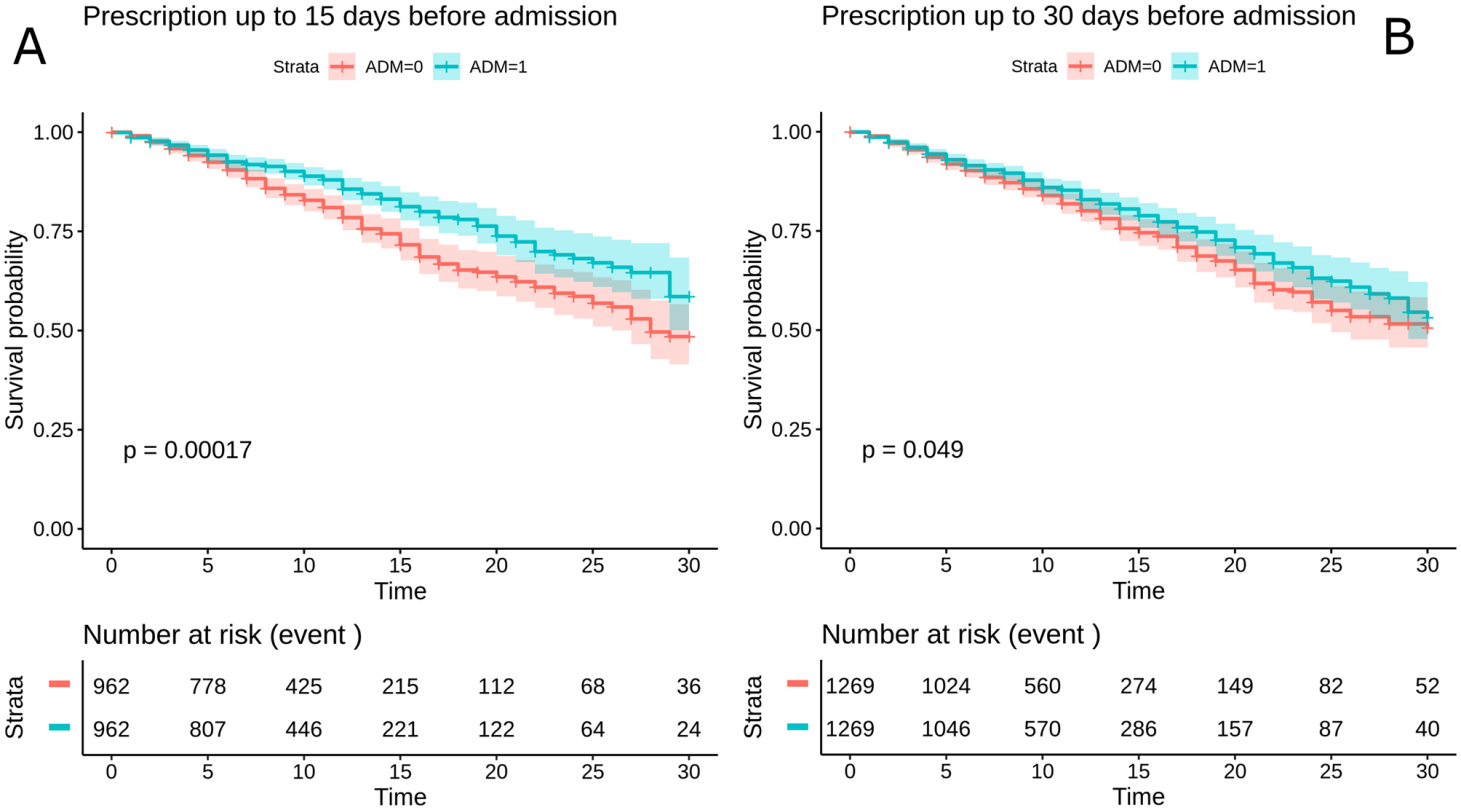
Kaplan–Meier curves of patients who received a prescription for ADM (**A**) 15 days and (**B**) 30 days before hospitalization for death outcome.

The effect of cholecalciferol, calcifediol or calcitriol prescription, both aggregated (ADM) and independently, 15 and 30 days prior hospitalization, was studied with respect to the outcome of death at 30 days. As described in “Methods”, PSM was applied to the treated and untreated patients. This rendered a satisfactory covariate balance and no significant correlations between the covariates was observed in the samples paired by the PSM model (Table 2). Kaplan–Meier curves shows the survival of patients who received a prescription for ADM 15 days (Fig. 1A) and 30 days (Fig. 1B) prior hospitalization, suggesting a significant association between ADM prescription and patient survival. Kaplan–Meier curves for specific cholecalciferol, calcifediol or calcitriol prescriptions (Fig. S2) supporting the same significant association between any of the individual prescriptions and patient survival, except for calcitriol with an erratic (non-significant) behavior due the already mentioned small sample size. The comparison of specific prescriptions supports a significantly increased survival of patients who received a prescription for calcifediol than those who received a prescription for cholecalciferol (see Table 3), pointing to a stronger association of calcifediol with patient survival.

**Table 2:** Matched covariates across treated and untreated patients. ADM columns contain the absolute number of treated and untreated and the percentage between parentheses. The column p-value corresponds to a χ^2^ test that systematically demonstrates that the values are equilibrated between both groups (no significant differences between ADM treated and untreated).

Days	Covariate	ADM treated	ADM untreated	p-value
30	Total N	1269	1269	
30	Sex (female)	766 (60.4)	769 (60.6)	0.9
30	Flu vaccine	722 (56.9)	714 (56.3)	0.8
30	Pneumococcal vaccine	491 (38.7)	505 (39.8)	0.6
30	Obesity	248 (19.5)	266 (21.0)	0.4
30	Hypertension	882 (69.5)	881 (69.4)	1.0
30	Chronic heart diseases	445 (35.1)	453 (35.7)	0.8
30	Cancer	215 (16.9)	210 (16.5)	0.8
30	Chronic digestive diseases	253 (19.9)	254 (20.0)	1.0
30	Chronic pulmonary diseases	309 (24.3)	292 (23.0)	0.5
30	Dementia	158 (12.5)	148 (11.7)	0.6
30	Diabetes	439 (34.6)	450 (35.5)	0.7
30	Asthma	167 (13.2)	158 (12.5)	0.6
30	**Age_bin**			0.3
30	01_41	69 (5.4)	85 (6.7)	
30	41_68	408 (32.2)	385 (30.3)	
30	68_99	792 (62.4)	799 (63.0)	
15	Total N	962	962	
15	Sex (female)	566 (58.8)	576 (59.9)	0.7
15	Flu vaccine	525 (54.6)	510 (53.0)	0.5
15	Pneumococcal vaccine	342 (35.6)	312 (32.4)	0.2
15	Obesity	188 (19.5)	168 (17.5)	0.3
15	Hypertension	647 (67.3)	653 (67.9)	0.8
15	Chronic heart diseases	306 (31.8)	330 (34.3)	0.3
15	Cancer	153 (15.9)	162 (16.8)	0.6
15	Chronic digestive diseases	183 (19.0)	175 (18.2)	0.7
15	Chronic pulmonary diseases	224 (23.3)	231 (24.0)	0.7
15	Dementia	114 (11.9)	132 (13.7)	0.2
15	Diabetes	322 (33.5)	327 (34.0)	0.8
15	Asthma	128 (13.3)	125 (13.0)	0.9
15	**Age_bin**			0.1
15	01_41	58 (6.0)	65 (6.8)	
15	41_68	332 (34.5)	290 (30.1)	
15	68_99	572 (59.5)	607 (63.1)

**Table 3:** Comparison between the survival curves by the Log Rank test with the corresponding p-values both unadjusted and FDR-adjusted.

Days	Metabolite	Calcifediol (FDR-adjusted)	Cholecalciferol (FDR-adjusted)	Calcitriol (FDR-adjusted)	Calcifediol (unadjusted)	Cholecalciferol (unadjusted)	Calcitriol (unadjusted)
30	Cholecalciferol	0.1	–	–	0.06	–	–
30	Calcitriol	0.1	0.4	–	0.07	0.3	–
30	Untreated	0.03*	0.4	0.5	0.005**	0.3	0.5
15	Cholecalciferol	0.2	–	–	0.2	–	–
15	Calcitriol	0.1	0.1	–	0.05	0.07	–
15	Untreated	0.002**	0.02*	0.4	0.0003**	0.005**	0.4

* p < 0.05; ** p < 0.01.

**Figure 2:**
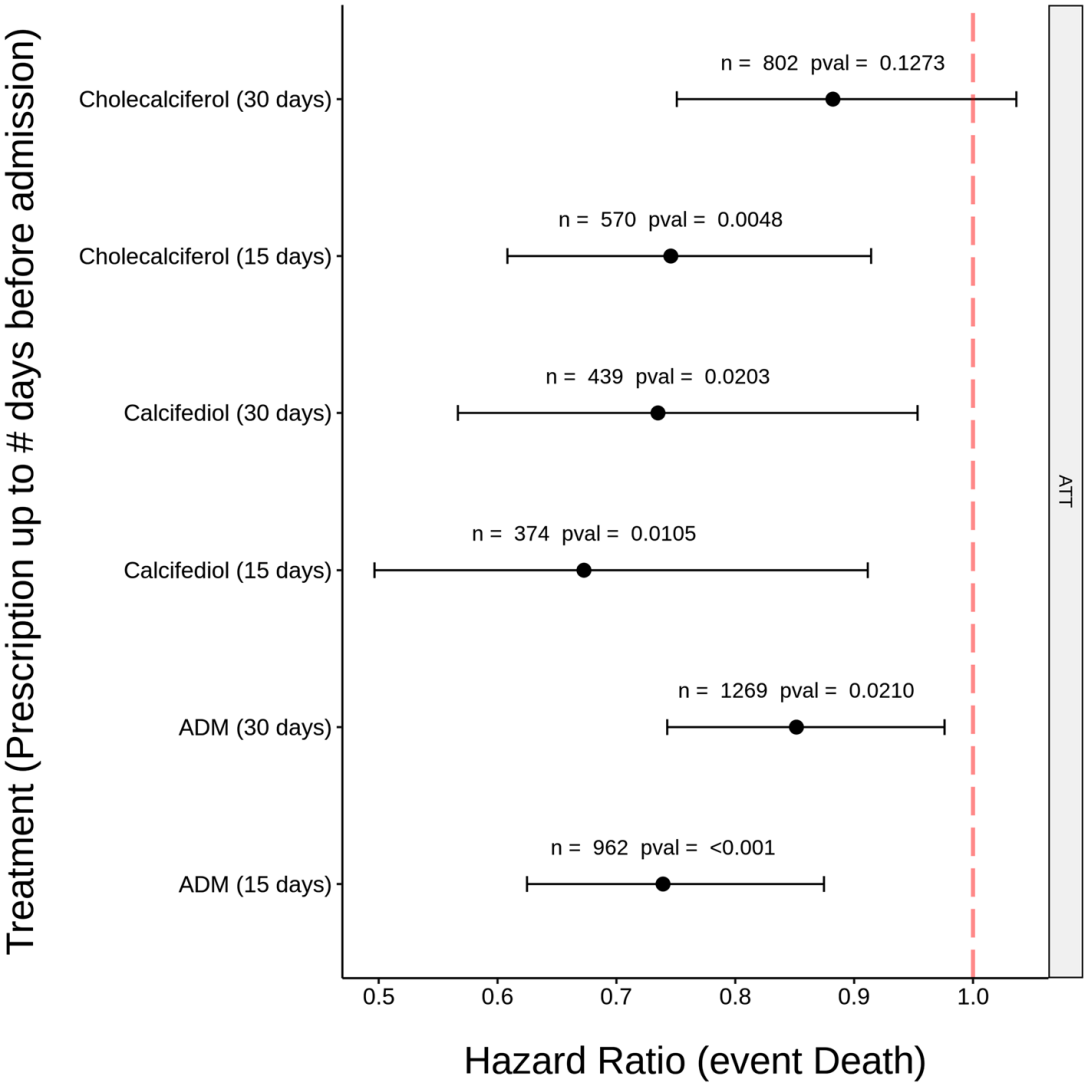
Hazard ratios with respect to the outcome death under ATT assumption for the cholecalciferol, calcifediol and ADM prescription in the two periods considered (15 and 30 days before hospitalization). In all the cases (except for cholecalciferol at 30 days) a significant association between prescription of these metabolites and patient survival is observed: confidence intervals do not cross the 1 line (α = 0.05).

To study in more detail the protective effect of VDES prescription a Cox regression was used to estimate the degree of association between the prescriptions and death risk by means of the hazard ratios. Figure 2 summarizes the hazard ratios with respect to the outcome death for the calcifediol and cholecalciferol prescriptions as well as the ADM prescription aggregated, in the two periods of administration considered (15 and 30 days prior hospitalization). Except for the case of cholecalciferol at 30 days, the prescriptions demonstrated a significant association with increased patient survival. From Fig. 2 it becomes apparent that calcifediol (HR = 0.67, CI [0.50–0.91]) shows a clearly higher association with patient survival than cholecalciferol (HR = 0.75, CI [0.61–0.91), when prescribed 15 days prior to hospitalization. However, if a larger period of 30 days is considered this effect decreases (calcifediol HR = 0.73, CI [0.57–0.95] and cholecalciferol HR = 0.88, CI [0.75, 1,03]). Calcitriol was not included, given the small sample size (< 30 patients). Additional analysis with different assumptions and different methodologies, such as bootstrap) support the results obtained (Fig. S3). Additionally, a more sophisticated analysis has been carried out to determine in detail the effect of each prescription along time (RMSTs). The RMST curve represents the expected survival days (on average) that subjects from the prescription group have with respect to untreated patients during the hospitalization time considered. Figure S4 shows that calcifediol prescription shows a significantly better survival than untreated for most of the 30 days interval studied. Cholecalciferol prescription shows better survival than untreated patients as well, although it is only statistically significant for a short period, and is always below calcifediol.

## Discussion

Andalusia, with 8.5 million inhabitants is the third largest region in Europe, having a population similar to Austria and being bigger than half of the countries of the European Union. Moreover, it has the largest population under a universal EHR. All data recorded by the Andalusian Public Health System is stored in the BPS. This allowed an unprecedented region-wise cohort study of 15,968 patients, which corresponds to all the COVID-19 patients that were hospitalized between January and November 2020.

This large-scale RWE study clearly shows that prescription for whatever indication with VDES metabolites significantly reduces the risk of death in patients hospitalized for COVD-19, This effect is stronger in patients who received a prescription for calcifediol, but also occurs in patients who received a prescription for cholecalciferol and inconclusive in the case of calcitriol (due to the small sample size its effect was not statistically significant). To our knowledge, this is the first study to investigate the effect of prior prescription of VDES metabolites (cholecalciferol, calcifediol or calcitriol) on death in patients hospitalized for COVID. Moreover, due to the country-size scale of this observational study it is easy to mimic the randomization element of a randomized clinical trial (RCT) and properly compare treatment groups, given the number of individuals available to properly adjust for all baseline cofounders^26^. Actually, the use of propensity scores provides additional adjustment to control for confounding variables^27^. Here, as shown in Table 2, confounding effects between the compared groups due to the known variables associated to the outcomes considered can be ruled out. The Health record system can only identify subjects receiving a prescription but cannot verify the actual intake of the prescribed drugs. Compliance is usually highest shortly after the prescription and declines afterwards. Therefore, due to the short time between the prescription and start of the COVID-19 infection it is highly likely that most subjects have taken their vitamin D (metabolites). A lower compliance may even imply a relative higher efficacy as the number of prescriptions and number of COVID-19 hospitalizations are well documented.

The consistency of the results presented here strongly suggests that patients who have been prescribed treatment preferentially with calcifediol (and less intensively with cholecalciferol) for other health objectives (usually for the prevention or treatment of primary or secondary osteoporosis or population at risk of vitamin D deficiency) presented a better response to COVID-19. In Andalusia, 25OHD deficiency (≤ 20 ng/ml) is usually very prevalent in spring and winter (34.5 and 37.2%), which is maintained even in summer (26.9%), but was probably higher during the lockdown in the period covered by the present study. 25OHD due to its half-life of two to three weeks is used as a nutritional marker of endocrine system levels of vitamin D and is a substrate for the synthesis of 1,25(OH)2D in multiple organs and systems^28^. However, the data used in this study did not contain information on the real doses of vitamin D or calcifediol taken prior the hospitalization. Therefore, it is unclear whether the better protection by calcifediol prescription compared to vitamin D itself is due to their intrinsic differences or was caused by different dosing.

Recently, numerous epidemiological and association studies have been published investigating the links between circulating levels of 25OHD, and the incidence and severity of COVID-19 infections^9–12,29^. Initially, these were observational ecologic studies that described a higher incidence of COVID-19 infection and death in countries where vitamin D deficiency (or low sun exposure) was common^12,30–32^. Thereafter, several studies evaluated the association between vitamin D status and risk or severity of SARS-CoV-2 infection^4–9^. Two recent meta-analyses have looked at published data linking vitamin D status with the risk or severity (ICU admission and mortality) of COVID-19 infections^12,13^. Probably related to the selection of studies they came to different conclusions, as one meta-analysis^12^ found an uncertain trend and the other^13^ a significantly increased risk of infection in subjects with the poorest vitamin D status. More relevant to the present study, one meta-analysis concluded that there was a non-significant trend between low 25OHD levels (below 20 ng/ml) and need for ICU admission, length of hospital stay or mortality^12^. The other meta-analysis with a greater restriction of high-quality studies, which included 2,756 patients, found a significantly higher mortality (OR: 2.47, 95% CI: 1.50 to 4.05), higher rates of hospital admissions (OR: 2.18, IC del 95%: 1.48–3.21 and longer hospital stay in subjects with the poorest vitamin D status^13^.

These results suggest that improving serum 25OHD concentration may improve the prognosis of COVID-19^11,12,29^. In a recent RCT, patients with baseline serum 25OHD levels < 20 ng/ml were compared with two standard treatment regimens in Spain (calcifediol 266 μg/month and cholecalciferol 25,000 IU (625 µg/month).—At first month, 13.5% (95% CI 9.1–19.0%) of patients in the calcifediol group and none of those receiving cholecalciferol (95% CI 0.0–3.7%) reached serum levels of 30 ng/ml. At month 4, 35. 0% of postmenopausal women treated with calcifediol and 8.2% of those treated with cholecalciferol reached serum 25(OH)D levels above 30 ng/ml (p < 0.0001). Few studies have compared the efficacy of calcifediol and cholecalciferol in increasing serum 25(OH)D levels and have reported similar findings. However, none of them compared monthly doses of both metabolites^33^.

A pilot controlled trial reported that administration of calcifediol versus no calcifediol reduced the need for ICU treatment in 76 hospitalized participants with COVID-19 who also received best available therapy^34^, as demonstrated initially by a pilot controlled trial in 76 hospitalized participants with COVID-19 who also received best available therapy which reported that administration of calcifediol 0.532 mg on entry and then 0.266 mg on day 3, 7, 14, 21, and 28) versus no calcifediol showed a dramatic decrease in the need for ICU admission in the treatment group (1 out of 50, 2%) as compared to the control group (13 out of 26, 50%). In order to determine if this difference was due to different characteristics of the patients in the two groups, the authors reported statistics for 15 prognostic risk factors and used a multivariate logistic regression to compute the adjusted odds ratio correcting for the two risk factors, hypertension and type 2 diabetes mellitus, that were significantly higher in the control group. After correcting for these imbalances, ICU admissions were still dramatically lower among the treated patients (odds ratio 0.03, 95% CI 0.003–0.25). The mortality rate among treated patients was also lower (0 out of 50 treated patients, 0%, versus 2 out of 26 control patients, 8%). The number of deaths was too small to achieve statistical significance against a null hypothesis of no effect, but the result is consistent with the plausible hypothesis that the decrease in mortality would be similar to the decrease in ICU admissions^34^. Recently, a larger observational cohort study included patients admitted to COVID-19 wards of Hospital del Mar, Barcelona, Spain. Calcifediol treatment using a similar schedule as in pilot study mentioned above, at hospital admission significantly reduced the need for ICU support and reduced mortality^35^. Out of 838 patients, 447 received calcifediol, whereas 391 were not treated at the time of hospital admission. The prescription of calcifediol was based on the ward they were assigned to, based on availability of beds. In five out of 8 wards patients received calcifediol whereas this was not the case in the other 3 wards. Treatment was otherwise similar and there were no significant baseline differences in patient characteristics. Among those treated on admission with calcifediol, 4.5% required ICU admission versus 21% in the untreated group. Logistic regression of calcifediol treatment on ICU admission, adjusted by age, gender, linearized 25OHD levels at baseline, and comorbidities showed that treated patients had a reduced risk to require ICU (OR 0.13, 95% CI 0.07–0.23). Moreover, 4.7% treated 55 with calcifediol at admission died compared to 15.9% of non-treated. Adjusted results showed a reduced mortality risk with an OR 0.21 [95% CI 0.10; 57 0.43]. In addition, in a retrospective study reported of patients hospitalized for laboratory confirmed COVID-19 infection, patients from five hospitals in Southern Spain received or not calcifediol (similar schedule mentioned formerly). Patients from one hospital received the option to receive calcifediol whereas this option was not available in the other hospitals. General treatment was otherwise very similar. In-hospital mortality during the first 30 days was 17.5%. The OR of death for patients receiving calcifediol (mortality rate of 5%) was 0.22 (95% CI 0.08–0.61), compared to patients not receiving such treatment (mortality rate of 20%; p = 0.000485). In the multivariable logistic regression model, there were significant differences in mortality for patients receiving calcifediol, compared with patients not receiving (OR = 0.104, 95% CI 0.027–0.404)^36^.

A multicenter, double-blind, randomized, placebo-controlled RCT in Brazil using a single large bolus administration of vitamin D3 (200,000 IU), showed however no improvement in outcome of COVID-19 (risk of death, need for mechanical ventilation or risk of ICU admission). Hospital stay was also not different between treated and untreated patients (mean 7 days in both). The start of vitamin D3 administration was very late in the evolution of the natural history of the disease (mean 10 days from the onset of symptoms), and 90% of all patients required oxygen at the start of the study^37^.

In a retrospective cohort study in the Barcelona area the risk of COVID-19 infection was evaluated in subjects who were prescribed vitamin D or calcifediol during the previous 4 months^38^. Similar to our study, the data were generated from the health records of the area. The hazard ratio of infection was slightly (but significantly) lower in subjects on vitamin D (HR = 0.95, CI 0.91–0.98) but not in subjects on calcifediol. Serum concentrations of 25OHD were available for a small subset of the population.

In assessing COVID-19 Outcomes in patients with serum 25OHD levels > 30 ng/mL, supplemented with cholecalciferol, they observed: that the rate of SARS-CoV2 infection (HR = 0.66, 95% CI 0.57–0.77), the risk of severe COVID-19 (HR = 0.72,  95% CI 0.52–1.00) and COVID-19 mortality (HR = 0.66, 95% CI 0.46–0.93) were significantly lower compared to vitamin D-deficient patients (25OHD < 20 ng/mL) not receiving supplementation. Similarly, when the treatment administered was calcifediol, both the rate of SARS-CoV2 infection (HR = 0.69, 95% CI 0.61–0.79), and the risk of severe COVID-19 (HR = 0.61, 95% CI 0.46–0.81 ) and most notably COVID-19 mortality (HR = 0.56, 95% CI (0.42–0.76) were significantly lower. This study had a different design than ours as we only included subjects with a recent (15–30 days) prescription of vitamin D or calcifediol (rather than supplementation in the previous 4 months) and also included subjects with severe infection requiring hospitalization.

The same research group in a retrospective cohort study in Catalonia (Spain), in patients with advanced chronic kidney disease (stages 4 and/or 5) evaluated the impact of calcitriol, the hormonal form of VDES. After propensity score matching, 6252 patients on calcitriol and 12,504 matched control patients were included in the study. Prescription of calcitriol was associated with a reduced risk of SARS-CoV2 infection (HR = 0.78, 95% 0.64–0.94), reduced risk of severe COVID-19 and reduced COVID-19 mortality (HR = 0.57, 0.41–0.80). In treated patients, regardless of renal function, there was an inverse association between mean daily dose of calcitriol and COVID-19 severity or mortality^39^. Supporting these general observations another recent study shows the association between vitamin D deficiency and higher risk of COVID-19 hospitalization^40^.

From a mechanistic perspective, there is good reason to postulate that the vitamin D endocrine system may have beneficial effects on different stages of COVID-19 infections such as the early viral infection (by innate immunity antiviral effector mechanisms, including induction of antimicrobial peptides and autophagy) and the later hyperinflammatory phase of COVID-19^41–43^. Moreover, activation of the vitamin D receptor (*VDR*) signaling pathway may have a critical modulatory role to host responses in acute respiratory distress syndrome^42^ by decreasing the cytokine/chemokine storm, producing a shift towards amplified adaptive Th2 immune responses, regulating the renin-angiotensin-bradykinin system, modulating neutrophil activity^44^ and maintaining the integrity of the pulmonary epithelial barrier, stimulating epithelial repair^45–47^ and decreasing the increased coagulability and prothrombotic tendency associated with severe COVID-19^41,42,48,49^. Regulation of the renin-angiotensin-bradykinin system is of particular relevance in mitigating the progression of severe COVID-19, where over-activation of RAS is associated with a poor prognosis^50^. Moreover, the protective effect of drugs targeting the *VDR* and the *GC* (Vitamin D Binding Protein or *DBP*) proteins of VDES has been suggested in a recent study on systematic drug repurposing for COVID-19^16^. The ML study has demonstrated the relevance of drugs targeting *VDR* and *GC* (*DBP*) proteins in the activity of COVID19-related signaling circuits (see Table S1). These signaling circuits affect cellular processes involved in modulating the immune activity, decreasing the inflammatory response, but also in slowing down cellular energetics.

Thus, both observational evidence and mechanistic knowledge support a crucial role of the vitamin D endocrine system in the response to severe outcomes of the COVID-19.

## Conclusions

This study strongly suggests that calcifediol or cholecalciferol prescriptions established previously to hospitalization were associated with a better survival rate among hospitalized COVID-19 patients. A significant reduction in mortality between 10 and 50% is observed when the prescription of cholecalciferol and calcifediol, respectively, was made within 15 days prior to hospitalization, and from 5 to 43% if the period considered for vitamin D prescription expands to 30 days. Most likely this effect occurs through *VDR* stimulation. VDES metabolite treatment may represent an effective, accessible, safe, well-tolerated and cost-effective preventive therapeutic approach for COVID-19, which is dramatically increasing in incidence and for which few validated treatments currently exist. Further large prospective, preferably interventional, Randomized Controlled Trials are needed to confirm whether regular treatment or supplementation of older adults with calcifediol or vitamin D3 improves COVID-19 outcomes.

The results reported here support the establishment of public health policies that make it possible to maintain adequate levels of 25OHD for the synthesis of calcitriol to enable a better prognosis in patients affected by COVID-19. In the light of the results obtained, calcifediol preferably, or cholecalciferol with a lower effect, can adequately meet these objectives. In fact, calcifediol may have some advantages over native vitamin D3. Thus, the former has a more reliable intestinal absorption (close to 100%) and can more rapidly restore serum concentrations of 25OHD as it does not require hepatic 25-hydroxylation^33,51^. In fact, calcifediol is three times more potent than oral cholecalciferol in raising serum 25OHD levels^52^. This is especially relevant in clinical situations whereby rapid restoration of serum 25OHD is desirable and *CYP2R1* expression is compromised^52^, explaining and giving causal consistency to the stronger association between survival and the prescription of calcifediol fifteen and thirty days prior to hospitalization by COVID-19.

This cost-effective and widely available treatment could have positive implications for the management of COVID-19 worldwide, particularly in developing countries.

## Materials and methods

### Design and patient selection

This study aimed to study a retrospective cohort including all Andalusian patients hospitalized with COVID-19 diagnosis. Following the inclusion criteria of having a COVID-19 diagnosis (by PCR or antigen test) and an overlapping hospitalization during the period January to November.

### Data preprocessing

Medication data related to VDES metabolites in the office and hospital pharmacy records were found for the following pharmaceutical compounds: cholecalciferol, calcifediol and calcitriol. For this study, individuals with prescriptions for a specific metabolite within a period from P days (P = 15 and 30 days) before the hospital admission until the discharge (or death) were compared to untreated individuals (with no prescriptions for VDES metabolites). In parallel, individuals were considered prescribed with All Vitamin D Metabolites (ADM) in the case that one of the previous pharmaceutical compounds were prescribed. A total of 570 patients received a prescription for cholecalciferol, 374 for calcifediol, and 18 for calcitriol if a period of P = 15 days is considered, and 802, 439 and 28, respectively, if a P = 30 days period is considered. Calcitriol was excluded from the individual studies, due to the low number of cases, but was considered as part of ADM, totaling 962 and 1269 for 15 and 30 days, respectively.

The main primary outcome was COVID-19 death (certified death events during hospitalization). Following previous similar studies, the first 30 days of hospital stay were considered for survival calculations^53^. The time variable in the models corresponds to the length (in days) of hospital stay. The stays that imply one or more changes of hospital units are combined in a single stay where the admission and discharge dates are set to either the start of the first or the end of the last combined stay. Finally, in order to reduce possible confounding effects due to reinfection mechanisms we have opted to include only the first stay for each patient.

### Propensity score matching

To reduce the confounding effects of several conditions on the outcome a 1:1 ratio Propensity Score Matching (PSM) was applied to match treated (in this case treatment refers to patients who received a prescription) and untreated patients without replacement in the survival analysis. Variables previously associated with COVID-19 mortality, such as: age, sex, pneumonia/flu vaccination status, hypertension, chronic obstructive pulmonary disease, diabetes, obesity, chronic pulmonary and digestive diseases, asthma, chronic heart diseases and cancer were included^54^ (Table 4). All the values agree with previous reports^55^. The propensity scores have been estimated by means of a Generalized Additive Model with a logit as the link function while the matching, to ensure a similar distribution of all the covariates across prescription groups, has been done using the nearest neighbor matching modality^56^. To ensure a similar distribution of all the covariates across the compared groups (beyond considering only standardized means differences) the higher order moments of covariates were used as recently recommended^57^. Covariate balance in matched samples is checked by a X^2^ test to confirm that no biases against prescribed or untreated matched patients exist.

**Table 4:** Variables previously associated to COVID-19 prognosis and symptoms in previous publications^54^ as distributed in the whole set of patients studied here. Columns survival and death contain the absolute number of individuals with the specific covariate, and in parentheses the percentage, that survive and die, respectively. A X^2^ test is carried out to check for direct associations (with no covariate correction) with death. The difference of percentages accounts for the effect: e.g. asthma protects and cancer increases the risk. The p-value column accounts for the significance.

Days	Covariate	Survival	Death	p-value
30	Total N	13,247	2706	
30	Asthma	1638 (12.4)	274 (10.1)	0.001
30	Flu vaccine	5437 (41.0)	1758 (65.0)	< 0.001
30	Pneumococcal vaccine	3467 (26.2)	1121 (41.4)	< 0.001
30	Obesity	2303 (17.4)	473 (17.5)	0.9
30	Hypertension	7518 (56.8)	2090 (77.2)	< 0.001
30	Chronic heart diseases	3333 (25.2)	1295 (47.9)	< 0.001
30	Cancer	1548 (11.7)	551 (20.4)	< 0.001
30	Chronic digestive diseases	2171 (16.4)	440 (16.3)	0.9
30	Chronic pulmonary diseases	2903 (21.9)	834 (30.8)	< 0.001
30	Diabetes	3838 (29.0)	1175 (43.4)	< 0.001
30	Dementia	958 (7.2)	536 (19.8)	< 0.001
30	Sex (female)	6088 (46.0)	1136 (42.0)	< 0.001
30	**Age_bin**			< 0.001
30	01_41	1587 (12.0)	22 (0.8)	
30	41_68	5945 (44.9)	395 (14.6)	
30	68_99	5715 (43.1)	2289 (84.6)	
15	Total N	13,258	2710	
15	Asthma	1643 (12.4)	274 (10.1)	0.001
15	Flu vaccine	5445 (41.1)	1761 (65.0)	< 0.001
15	Pneumococcal vaccine	3471 (26.2)	1123 (41.4)	< 0.001
15	Obesity	2308 (17.4)	474 (17.5)	0.9
15	Hypertension	7527 (56.8)	2094 (77.3)	< 0.001
15	Chronic heart diseases	3334 (25.1)	1299 (47.9)	< 0.001
15	Cancer	1548 (11.7)	553 (20.4)	< 0.001
15	Chronic digestive diseases	2174 (16.4)	440 (16.2)	0.9
15	Chronic pulmonary diseases	2909 (21.9)	834 (30.8)	< 0.001
15	Diabetes	3843 (29.0)	1176 (43.4)	< 0.001
15	Dementia	958 (7.2)	537 (19.8)	< 0.001
15	Sex (female)	6096 (46.0)	1140 (42.1)	< 0.001
15	**Age_bin**			< 0.001
15	01_41	1588 (12.0)	22 (0.8)	
15	41_68	5948 (44.9)	395 (14.6)	
15	68_99	5722 (43.2)	2293 (84.6)	

### Survival on the matched samples

Kaplan–Meier estimate was used to infer the survival probability difference between patients which received a prescription and untreated patients. Survival curves for the different groups are compared with a Log Rank test.

### Robust estimation of the treatment (vitamin D prescription) effect using the whole population

Although PSM is a widely used technique because it leverages the use of parametric and non-parametric models to covariate-treatment-outcome unbalanced data, the consistency of any estimator derived from the propensity scores is limited by exchangeability assumptions between the treated and untreated samples, the covariate adjustment and model specification (among others) mainly due to the fact that the propensity score is computed with the same data as the modelling. Here, the hazard ratios for each of the treatments of interest have been computed by means of the closed-form estimator^58^ using a weighted Cox model with inverse propensity weighting under the Average Treatment Effect on the Overall (ATE) and the Average Treatment Effect on the Treated (ATT) assumptions, the most used weighting approximations to estimate treatment effects^59^. Note that the ATE weights are stabilized by factoring the overall probability of being exposed to a given treatment into the equation^58^.

Furthermore, an alternative estimation of the treatment effect has been obtained by means of bootstrapping (n = 1000 iterations) a weighted Cox model with the sample weights computed by means of a Binomial General Linear Model which regress the treatment as a function of the covariates^60^.

### Modeling survival along time

For each time point the restricted mean survival time (RMST) of the treated versus the untreated for each prescription has been compared. The RMST is computed as the area under the survival curve up to the time point (*t*) and, therefore, the comparison measures the difference and ratio of RMST between treated and untreated patients. The interpretation of the curve is straightforward, representing each time point (*t*) the expected days (on average) that subjects from the group with vitamin D metabolite prescriptions live longer (or shorter) than untreated patients when patients are followed up to time *t*. Interestingly, the significance of the RMST comparison can be estimated (p-value corrected for multiple testing with False Discovery Rate, FDR^61^) for each time point. Note that the dynamic estimated ratio of RMST is more prone to detect plateaus on the treatment (vitamin D metabolite prescriptions) effect over time^62^, so both curves are complementary.

### Software

For the matching analysis we have used the *MatchIt*^63^ R package (version 4.1.0). The treatment effect models have been implemented with the *hrIPW*^64^ R package (version 0.1.3). RMST computations have been performed with the survRM2^65^ R package (version 1.0.3). Survival curves and plots have been generated with the R *survival*^66^ (version 3.2.7) and *survminer*^67^ (version 0.4.8) packages, respectively.

### Ethics declaration

The Ethics Committee for the Coordination of Biomedical Research in Andalusia approved the study “Retrospective analysis of all COVID-19 patients in the entire Andalusian community and generation of a prognostic predictor that can be applied preventively in possible future outbreaks” (29^th^ September, 2020, Acta 09/20) and waived informed consent for the secondary use of clinical data for research purposes. All research was performed in accordance with relevant guidelines and regulations.

## Supplementary Information

Supplementary Information.

## Data Availability

The data that support the findings of this study are available from the Andalusian Population Health Database (Base Poblacional de Salud: https://www.sspa.juntadeandalucia.es/servicioandaluzdesalud/profesionales/sistemas-de-informacion/base-poblacional-de-salud) but restrictions apply to the availability of these data, which were used under license for the current study, and so are not publicly available.
