# Supplementary material for: Real world evidence of calcifediol or vitamin D prescription and mortality rate of COVID-19 in a retrospective cohort of hospitalized Andalusian patients
Source: Sci Rep. 2021 Dec 3;11:23380. doi: 10.1038/s41598-021-02701-5 (PMC8642445; doi:10.1038/s41598-021-02701-5)
Supplement: Supplementary file 1 — Supplementary Information. [file 41598_2021_2701_MOESM1_ESM.pdf]

Real world evidence of calcifediol or vitamin D prescription and mortality rate of COVID-19 in a retrospective cohort of hospitalized Andalusian patients

Carlos Loucera, María Peña-Chilet, Marina Esteban-Medina, Dolores Muñoyerro-Muñiz, Román Villegas, Jose Lopez-Miranda, Jesus Rodriguez-Baño, Isaac Túnez, Roger Bouillon, Joaquin Dopazo*, Jose Manuel Quesada Gomez*

Supplementary material

Figure S1. Hospitalization dates of the patients included in the study along the period studies (January to November, 2020). The admission trend is clearly overlapping with the COVID-19 waves. Obviously, for a few early hospitalizations the diagnosis was posterior.

Figure S2. Kaplan-Meier curve for calcifediol, cholecalciferol and calcitriol for death outcome.

Figure S3. Bootstrapping (n=1000 iterations) of a Cox model with the propensity weights computed by means of a Binomial General Linear Model (GLM) which regress the treatment as a function of the covariates. The bootstrapping approximates the hazard estimations of the closed form under both estimands: ATE and ATT for outcome: death.

Figure S4. RMST curves for the three treatments. RMST represents the expected survival days (on average) that subjects from the treatment group have with respect to untreated patients along time both as number of days (upper panels) or as a ratio (lower panels) for both prescriptions 15 days before admission (right panels) and 30 days (left panels). Dots (triangles, squares or circles) in the curves correspond to time points with a significant RMST, FDR-adjusted p-value < 0.05.

Table S1. Signaling circuits found to be relevantly affected (according to the ML algorithm) by the *VDR* and *GC* genes, which are targets of calcifediol, cholecalciferol and calcitriol.

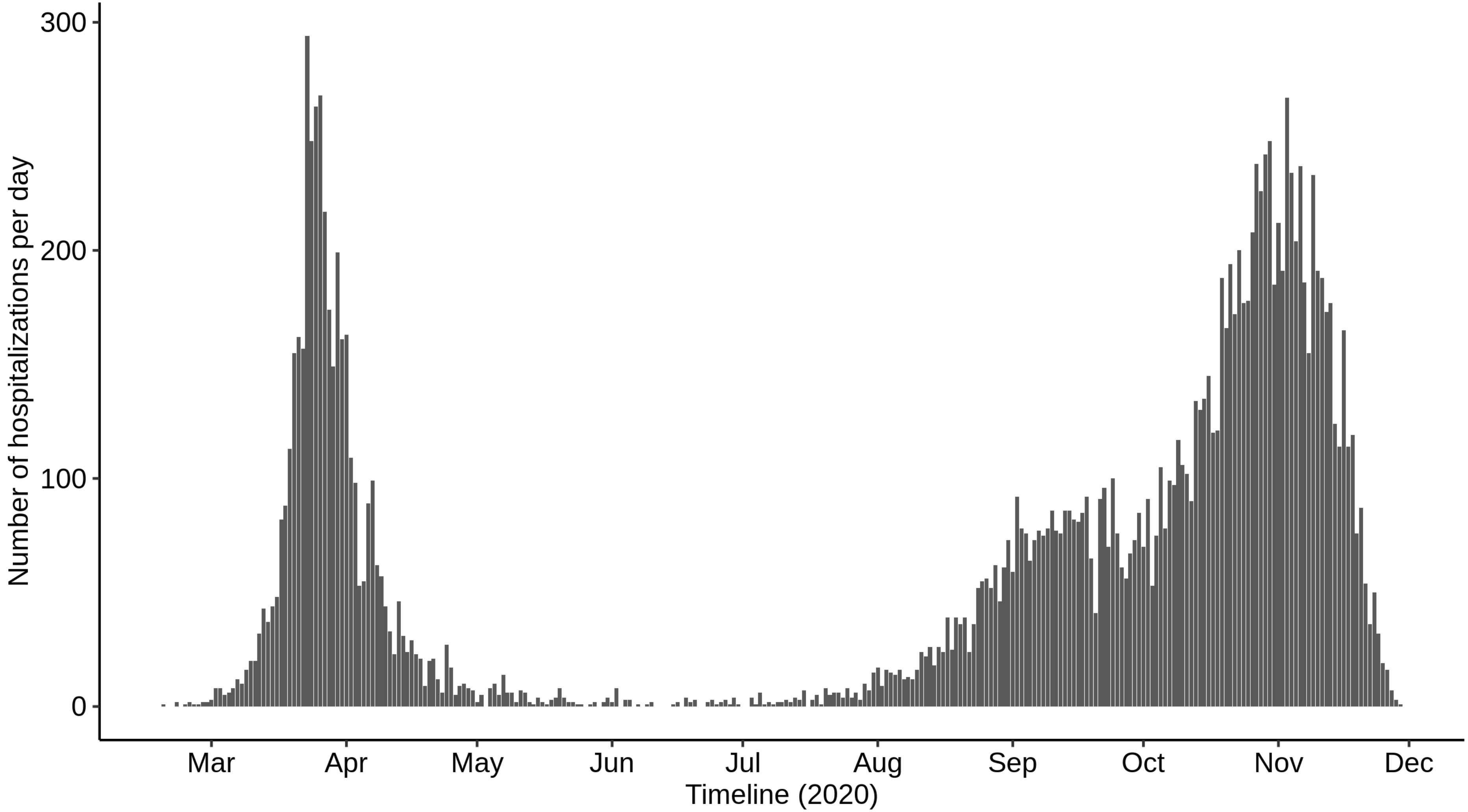

Figure S1. Hospitalization dates of the patients included in the study along the period studies (January to November, 2020). The admission trend is clearly overlapping with the COVID-19 waves. Obviously, for a few early hospitalizations the diagnosis was posterior.

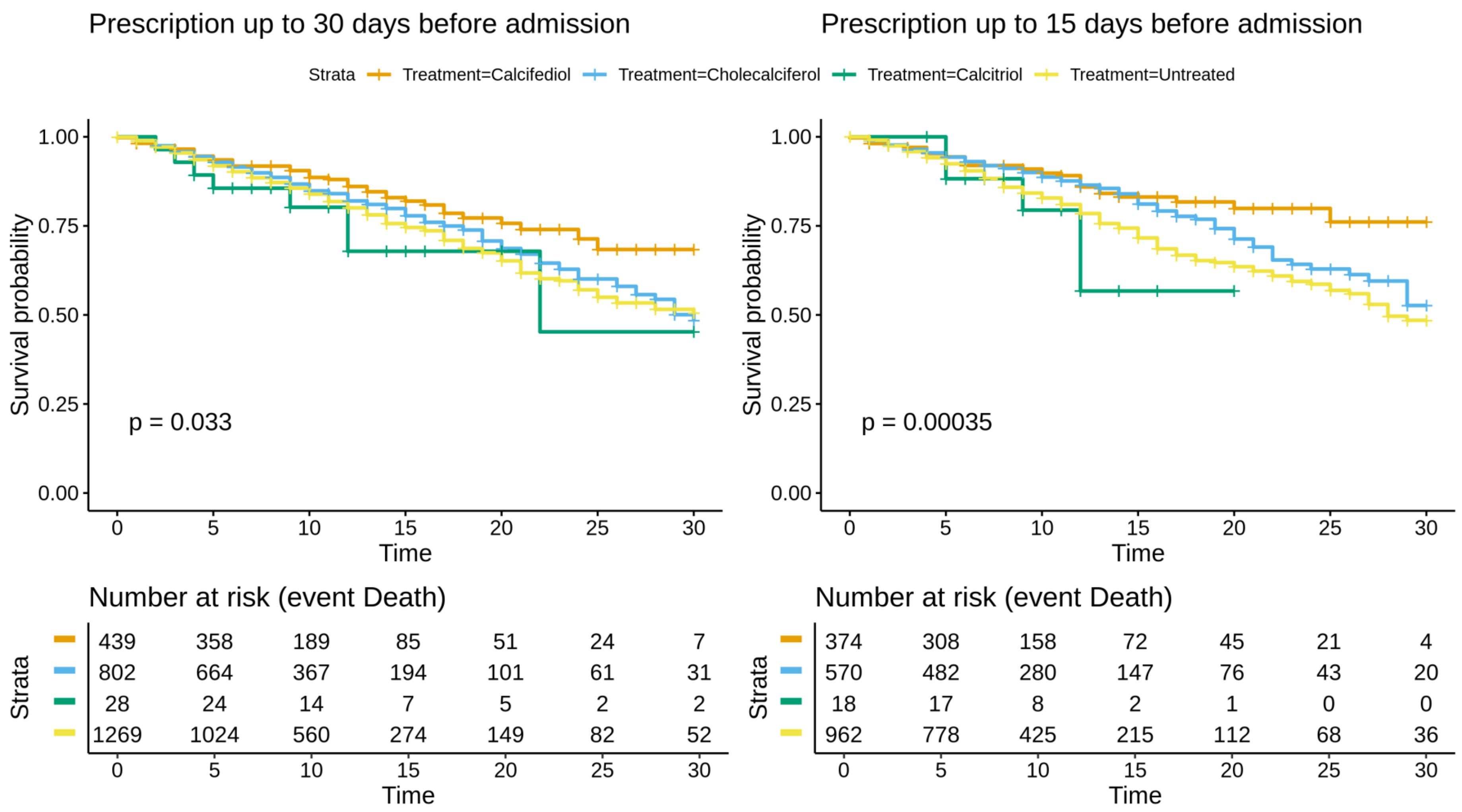

Figure S2. Kaplan-Meier curve for calcifediol, cholecalciferol and calcitriol for death outcome.

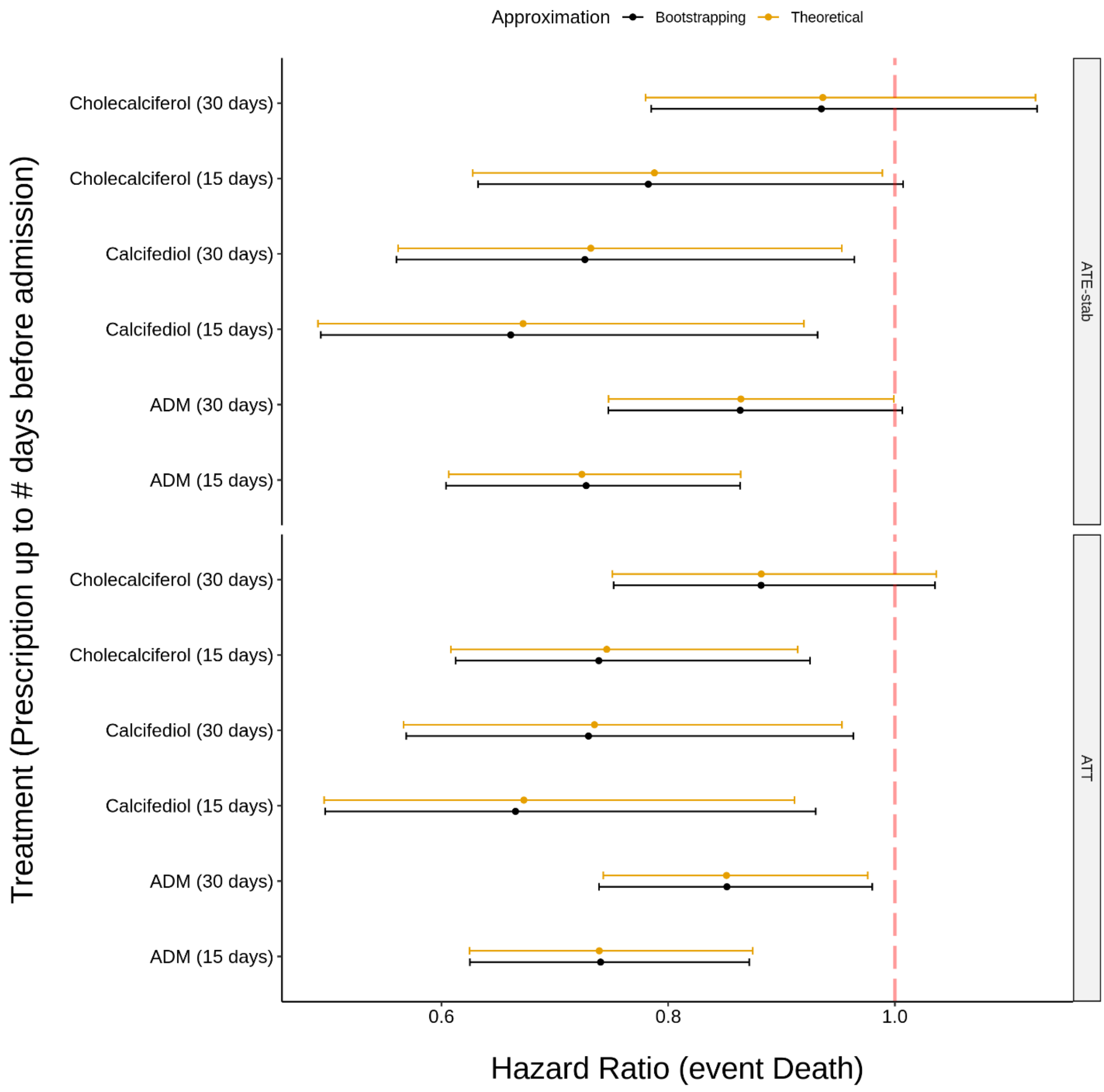

Figure S3. Bootstrapping (n=10000 iterations) of a Cox model with the propensity weights computed by means of a Binomial General Linear Model (GLM) which regress the treatment as a function of the covariates. The bootstrapping approximates the hazard estimations of the closed form under both estimands: ATE and ATT for outcome: death.

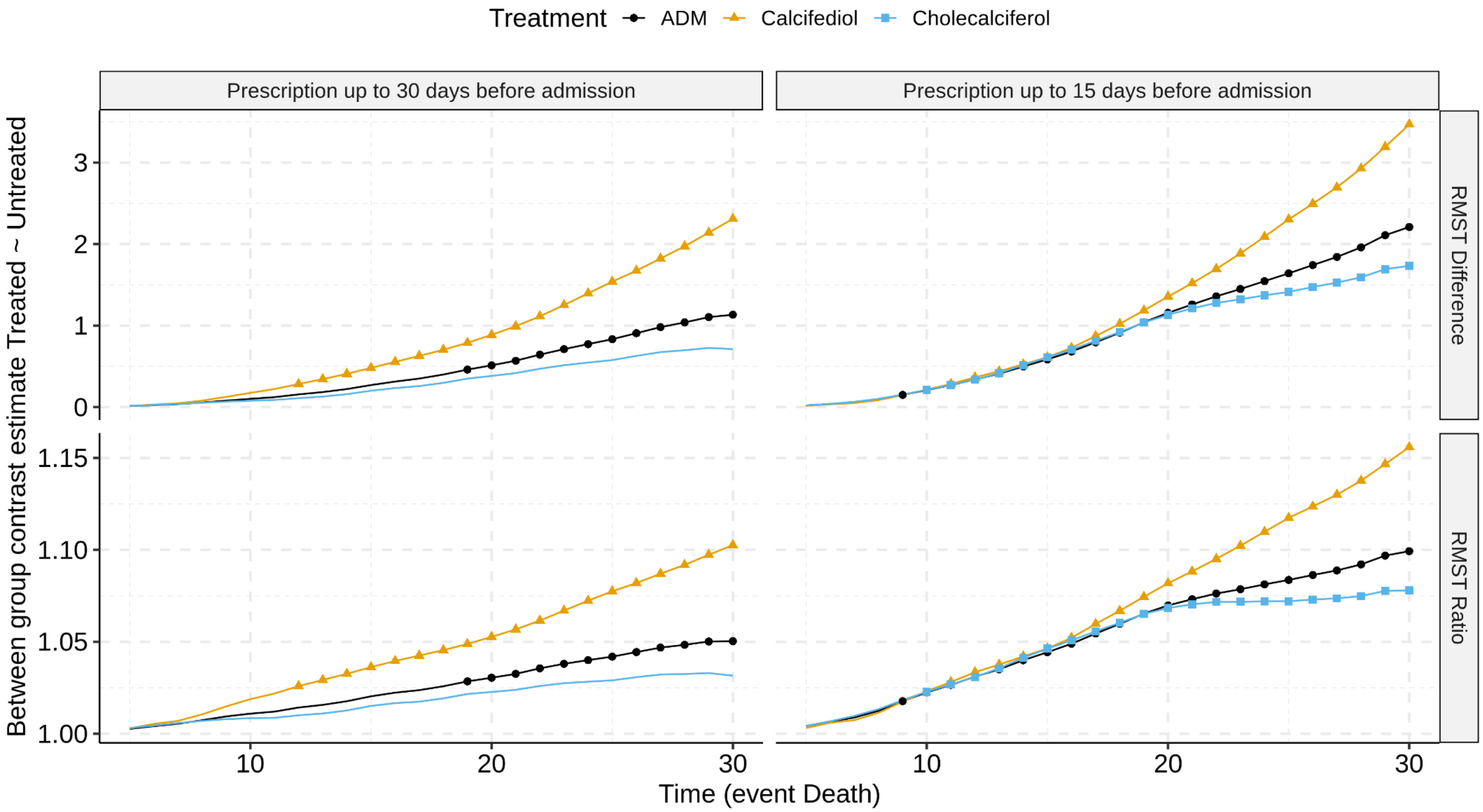

Figure S4. RMST curves for the three treatments. RMST represents the expected survival days (on average) that subjects from the treatment group have with respect to untreated patients along time both as number of days (upper panels) or as a ratio (lower panels) for both prescriptions 15 days before admission (right panels) and 30 days (left panels). Dots (triangles, squares or circles) in the curves correspond to time points with a significant RMST, FDR-adjusted p-value < 0.05.

Table S1. Signaling circuits found to be relevantly affected (according to the ML algorithm) by the *VDR* and *GC* genes, which are targets of calcifediol, cholecalciferol and calcitriol. Signaling circuits are sub-pathways defined within KEGG pathways. They are named as "pathway:gene", being the pathway to which they belong to and the effector gene at the end of the signal transduction circuit that triggers the function(s) defined by the GO terms and summarized as general COVID-19 hallmarks. These signaling circuits affect cellular processes involved in modulating the immune activity and decreasing inflammatory response, and also in slowing down the cellular energetics. All these processes have been described in COVID-19 and linked with a higher susceptibility to develop severe symptoms.

**Circuit**	***VDR* relevant**	***VDR* score**	***GC* relevant**	***GC* score**	**COVID-19 Hallmarks**	**GO function (from UniProt)**
Complement and coagulation cascades: *C2*	-	-	Y	6,88E-04	Immune activity	Complement pathway, Innate immunity, Immunity
Inflammatory mediator regulation of TRP channels: *TRPM8*	-	-	Y	7,06E-04	Energetics, Inflammatory response	Ion channel, Ion transport, Transport, Sensory transduction
Insulin signaling pathway: *PKLR*	-	-	Y	2,50E-04	Energetics	Glycolysis
Insulin signaling pathway: *GYS1*	-	-	Y	1,04E-04	Energetics	Gluconeogenesis
Adipocytokine signaling pathway: *PTPN11*	Y	5,98E-04	-	-	Inflammatory response	abortive mitotic cell cycle [GO:0033277]; activation of MAPK activity [GO:0000187]; cellular response to cytokine stimulus [GO:0071345]; epidermal growth factor receptor signaling pathway [GO:0007173]; fibroblast growth factor receptor signaling pathway [GO:0008543]; T cell co-stimulation [GO:0031295]
Adipocytokine signaling pathway: *POMC*	Y	5,51E-04	-	-	Immune activity, Anti-viral defense	antimicrobial humoral immune response mediated by antimicrobial peptide [GO:0061844]; cell-cell signaling [GO:0007267]; killing of cells of other organism [GO:0031640]
